# Mechanism of Transcription Factor ChbZIP1 Enhanced Alkaline Stress Tolerance in *Chlamydomonas reinhardtii*

**DOI:** 10.3390/ijms26020769

**Published:** 2025-01-17

**Authors:** Ao Wang, Rui Wang, Xiaoling Miao

**Affiliations:** 1 State Key Laboratory of Microbial Metabolism, School of Life Sciences and Biotechnology, Shanghai Jiao Tong University, Shanghai 200240, China; wangao199961@sjtu.edu.cn (A.W.); wang_rui@sjtu.edu.cn (R.W.); 2 Carbon-Negative Synthetic Biology for Biomaterial Production from CO2 (CNSB), Campus for Research Excellence and Technological Enterprise (CREATE), 1 CREATE Way, Singapore 138602, Singapore

**Keywords:** alkaline environments, transcription factor, microalgae, photosynthesis, fatty acid desaturation, antioxidant

## Abstract

Alkaline environments such as alkaline lands, lakes, and industrial wastewater are not conducive to the growth of plants and microorganisms due to high pH and salinity. ChbZIP1 is a bZIP family transcription factor isolated from an alkaliphilic microalgae (*Chlorella* sp. BLD). Previous studies have demonstrated its ability to enhance alkaline tolerance in *Arabidopsis thaliana*. However, the potential of ChbZIP1 to confer similar alkaline tolerance in other microalgae remains unclear, and the specific mechanisms are not fully understood. The analysis of cellular physiological and biochemical indicators revealed that the ChbZIP1 transformants exhibited enhanced photosynthetic activity, increased lipid accumulation, and reduced fatty acid unsaturation. Genes associated with cellular reactive oxygen species (ROS) detoxification were found to be upregulated, and a corresponding increase in antioxidant enzyme activity was detected. In addition, the relative abundance of intracellular ROS and malondialdehyde (MDA) was significantly lower in the transformants. In summary, our research indicates that ChbZIP1 enhances the tolerance of *Chlamydomonas reinhardtii* to alkaline environments through several mechanisms, including the repair of damaged photosynthesis, increased lipid accumulation, improved fatty acid unsaturation, and enhanced antioxidant enzyme activity. This study aims to contribute to a more comprehensive understanding of the mechanisms underlying alkalinity tolerance in microalgae and offers new insights and theoretical foundations for the utilization of microalgae in alkaline environments.

## 1. Introduction

The salinization of soil and water has become a worldwide environmental problem. According to estimates by the Food and Agriculture Organization (FAO), the global saline-alkali land area reached 1.03 billion hm^2^ in 2015, and it is still growing at a rate of about 1 million hm^2^ every year. High concentrations of CO_3_^2−^ and HCO_3_^−^ in saline-alkali soil (lake) result in severe stresses, which do great harm to the growth of plants and microorganisms as well as the ecological environment [1]. In addition to the saline-alkali soil (lake), the pH changes caused by the alkaline wastewater from human industrialization such as papermaking, beer and leather manufacturing can also directly affect the physiological functions of plants and their nutrient utilization [2]. If these alkali environments can be rationally developed and utilized, people’s needs for energy, food, and water resources will be effectively alleviated. However, only a few organisms such as alkali-tolerant plants, fungi, algae, and bacteria can survive in alkaline environments [3].

It has been reported that cells exposed to alkaline environments produce more reactive oxygen species (ROS, O_2_^−^, H_2_O_2_) compared with salt stress alone [4,5,6]. The high pH values in alkaline environments can directly destroy the structure and function of biological macromolecules, membranes, and organelles [7,8,9]. Especially in the cells of photosynthetic organisms, alkaline stress can damage the chloroplast structure, thereby affecting the photosynthesis of plants and causing the energy metabolism process to be blocked [10,11]. In addition, high pH values lead to a decrease in the solubility of metal ions such as Fe^3+^ and Mg^2+^, which limits the utilization of various inorganic metal ions [12]. Although there is still no conclusive conclusion on the mechanism of how alkaline environments affect the growth of plants or microorganisms, the damage to cells may be more serious and complex than other stress environments, making it of important research value.

Transcription factors are major regulators of plant abiotic stress responses. TFs are characterized as containing a DNA-binding domain (DBD), an oligomerization domain, and transcription regulatory domains. These proteins control the expression of multiple target genes by binding to specific DNA motifs in the promoter region [13]. Compared with specific functional proteins, the genetic engineering of one or several TFs is sufficient to improve the organism’s tolerance to adverse environments, making these transcription factors attractive targets for genetic engineering. Through genome-wide analysis, a large number of different transcription factors have been found in different species, such as MYB, bHLH, WRKY, bZIP, and NAC. These TFs are involved in regulating various abiotic environmental stress responses, such as drought, high temperature, and cold [14]. Some transcription factors have also been shown to play a very important role in tolerance to salt-alkali environment. The alkali tolerance of transgenic Arabidopsis was improved by the heterologous expression of the transcription factor GsnAC019 in soybeans [15]. The AP2/ERF family transcription factor GsERF71 and the ethylene response factor GsERF6 have also been reported to enhance plant tolerance to alkaline stress [16,17]. The accumulation of H_2_O_2_ in tobacco mutant plants that overexpressed the transcription factor WRKY39 was significantly lower than wild type under saline-alkali conditions [18]. The bZIP (basic leucine zipper) TF family is widely distributed in eukaryotes and is characterized by a highly conserved basic DNA-binding domain [19]. Members of bZIP TFs function in a variety of different biological processes, including cell elongation, organ and tissue differentiation, main root growth, flower development, seed maturation, plant senescence, light response, and abiotic stresses (such as cold, high salinity, drought) [20,21,22,23]. However, the role of bZIP TFs in cells’ response to alkaline environments remains to be explored.

Microalgae are photosynthetic microorganisms. They are one of the main oxygen producers and play an important role in the ecosystem [24]. In recent years, the use of microalgae for the development of unfavorable environments such as wastewater treatment has been extensively studied [25]. *Chlamydomonas reinhardtii* is a model organism with three sequenced genomes that can be genetically transformed and is regarded as the Arabidopsis of microalgae. Compared with other microalgae, the development of the genetic engineering toolbox for *C. reinhardtii* is much more complete, making it a good genetic engineering chassis [26,27]. *C. reinhardtii* can be used as a genetically engineered chassis for the bioremediation of unfavorable environments such as wastewater, efficiently utilizingits nutrients and producing algae biomass for different purposes, such as obtaining recombinant proteins and oils [28,29]. In addition, using *C. reinhardtii* to treat wastewater is also an effective method of biological carbon sequestration [30]. It has been reported that *C. reinhardtii* can accumulate more oil in alkaline environments, but its limited growth has hindered its use in these environments as an excellent genetically engineered chassis [31].

In this study, we used genetic engineering to heterologously express the bZIP family TF ChbZIP1 from alkaliphilic microalgae *Chlorella* sp. BLD in *C. reinhardtii* CC-849 to obtain a transgenic mutant strain. In alkaline environments at pH = 9 and pH = 9.5, we successfully improved the transformant’s tolerance to alkaline environments, which was reflected in shorter growth recovery cycles and higher biomass accumulation, implying its environmental remediation capabilities and resource utilization potential. Then, we further revealed the possible mechanism of ChbZIP1 to improve cells’ tolerance to alkaline environments, enriching the research on the role of TFs in biological response to adverse environments and providing a theoretical basis for further guiding the genetic engineering of transcription factors to improve crops’ tolerance to alkaline environments.

## 2. Results

### 2.1. Identification of the ChbZIP1 from Chlorella sp. BLD

We previously reported that a new alkali-resistant microalgae *Chlorella* sp. BLD was discovered in Aiding Saline-alkali Lake in Xinjiang, China. It can grow well at extremely high pH (pH > 10) and also has high lipid yield [32]. In another subsequent study, we used transcriptomics to compare the differences in gene expression of *Chlorella* sp. BLD under pH = 7 and pH = 10 and successfully screened several differentially expressed transcription factors. The heterogeneous expression of one of the potential TFs, ChbZIP1, in yeast and Arabidopsis was found to successfully improve their survival under an alkaline environment [33]. Therefore, we chose to further describe ChbZIP1 and explore its possible regulatory mechanisms by heterologous expression in the model *C. reinhardtii*.

**Figure 1:**
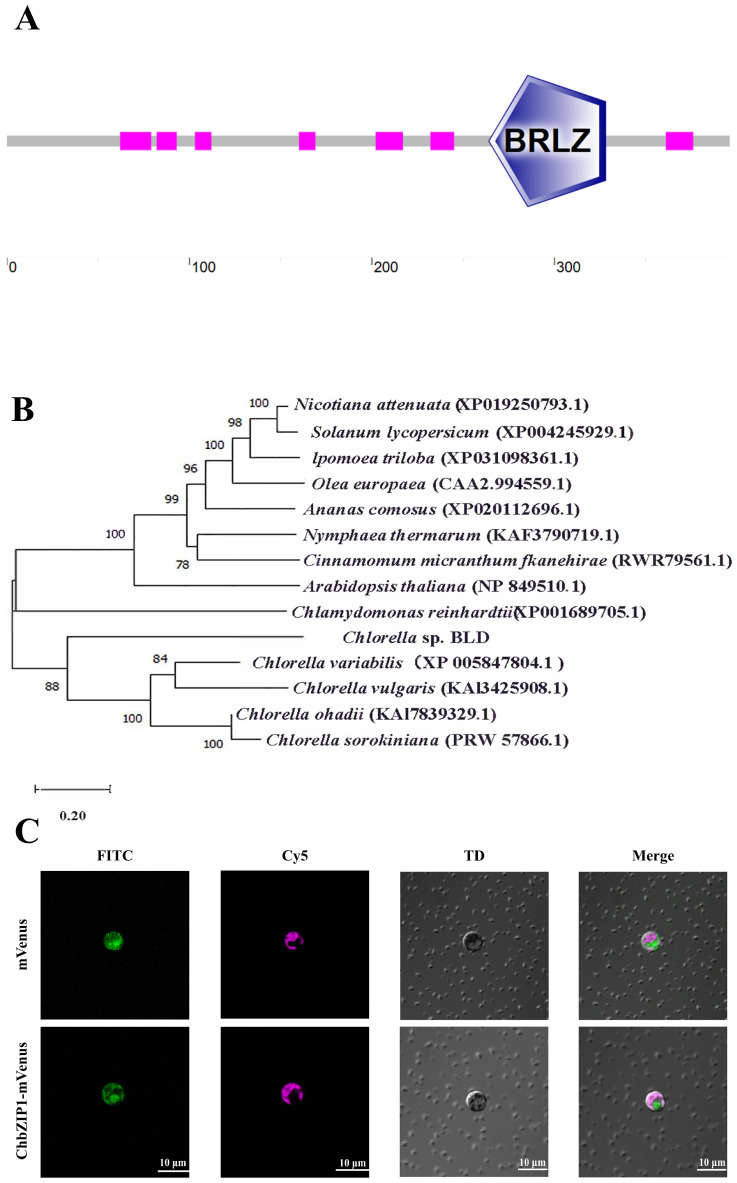
Sequence and phylogenetic analysis and the subcellular localization of ChbZIP1. (**A**) Schematic representation of ChbZIP1 showing the protein sequence with a basic leucine zipper (bZIP) domain. The purple pentagons represent the bZIP domains, and the red rectangles represent the low-complexity areas. (**B**) Neighboring phylogenetic relationships among ChbZIP1 and other species bZIPs. Chlorella BLD is shown in bold. (**C**) Subcellular localization of ChbZIP1 in *C. reinhardtii*. mVenus represents the fluorescent proteins; ChbZIP1-mVenus represents the ChbZIP1-mVenus fusion protein; red fluorescence represents the chloroplast autofluorescence of *C. reinhardtii*, green fluorescence represents the fluorescence of mVenus protein; bars = 10 μm.

The sequence analysis of ChbZIP1 showed that the ChbZIP1 protein contains a eukaryotic basic leucine zipper (bZIP) domain at the amino acid positions 264th to 328th. It contains a basic region that mediates sequence-specific DNA binding, followed by a leucine zipper region required for dimerization (Figure 1A).

The sequence alignment and neighbor-joining (NJ) tree analysis showd that the ChbZIP1 first aggregated with other chlorella on the tree, then with other microalgae and plants (Figure 1B). The subcellular localization of ChbZIP1 in the microalgae was explored using the model microalgae *C.reinhardtii*. The transformants expressing mVenus protein only showed fluorescence in the cytoplasm. However, the transformants expressing the fusion protein of mVenus and ChbZIP1 showed fluorescence in the cell nucleus (Figure 1C), which was consistent with the reported fluorescence observation results of the nuclear localization protein [34].

The above works show that ChbZIP1 is a basic leucine zipper protein localized in the nucleus and most likely has a transcriptional regulation function.

### 2.2. Heterogeneous Expression of ChbZIP1 Enhances Adaptability to Alkaline Stress in C. reinhardtii

To analyze the function of ChbZIP1, we optimized its sequence codons and inserted introns at specific locations to improve its expression efficiency (Supplementary Materials) [35]. Through transformation and screening, we obtained several transformants that successfully integrated ChbZIP1 into the genome and were able to be correctly clipped after expression (Figure S1). The foreign gene is randomly inserted into the genome of *C. reinhardtii*, which leads to differences in the expression efficiency among different transformants, and also affects the growth of *C. reinhardtii* [36]. Therefore, we selected two transformants (bZIP1-A7 and bZIP1-C16) that had no significant difference in growth from wild type (WT) under common TAP conditions for the next study.

**Figure 2:**
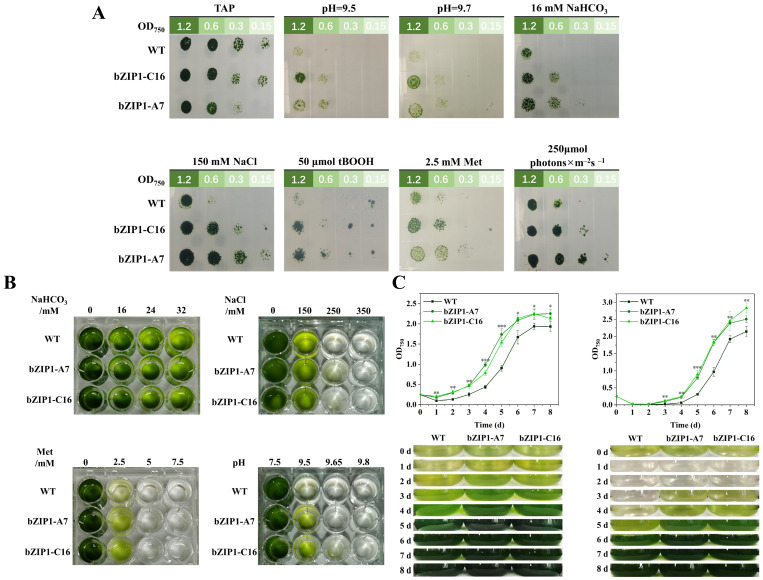
Growth of ChbZIP1-overexpressed transformants bZIP1-A7, bZIP1-C16, and WT under different stress conditions. (**A**) Agar plate growth analysis of the tolerance of transformants bZIP1-A7 and bZIP1-C16 to different stress environments compared to WT. Take the stock liquor into sterile EP tubes and dilute it 2/4/8 times, respectively. Then, take 10 μL of the stock liquor and the diluent, respectively, onto the plates set in various stress environments (pH = 9.5, pH = 9.7, 16 mM NaHCO_3_, 50 μmol t BOOH, 150 mM NaCl, 250 μ mol photons m^−2^s^−1^, and 2.5 mM Met), and take photos after 4 days. (**B**) A 24-well plate liquid medium growth analysis of the tolerance of transformants bZIP1-A7 and bZIP1-C16 to different stress environments compared to WT. Add the stock liquor into a 24-well plate liquid culture medium in various stress environments (pH = 7.5/9.5/9.65/9.8, 16/24/32 mM NaHCO_3_, 150/250/350 mM NaCl, 2.5/5/7.5 mM Met) at OD_750_ = 0.1, and take photos after 4 days. (**C**) Quantitative analysis of dynamic growth of bZIP1-A7, bZIP1-C16, and WT in an alkaline environment. They were first grown in a TAG medium at pH 7.0, and then transferred to an AMPSO/CHES-acetate phosphate medium (pH = 9.0/9.5) and cultured to the plateau. Representative photos of cultures for designated days were taken (bottom picture) and the biomass for the corresponding days was determined (top picture). WT represents wild-type algal strain; bZIP1-A7 and bZIP1-C16 represent transformants with the overexpression of ChbZIP1. Values are mean ± SD, (*n* = 3). Student’s *t*-tests were performed to determine statistically significant differences: *, *p* < 0.05; **, *p* < 0.01; and ***, *p* < 0.001.

In order to explore the adaptability of ChbZIP1 transformants to various abiotic stresses, the growth of wild-type (WT) *C. reinhardtii* CC-849 and ChbZIP1 transformants (bZIP1-A7 and bZIP1-C16) in normal and various stress conditions (pH = 9.5, pH = 9.7, 16 mM NaHCO_3_, 150 mM NaCl, 2.5 mM Met, 50 μmol tBOOH and 250 μmol photons m^−2^ s^−1^) on agar plates was studied after 4 days. The results showed that there was almost no difference in the growth of transformants and WT in the normal conditions. In stress conditions, the growth of the two transformants was better than WT (Figure 2A). This indicated that ChbZIP1 may improve *C. reinhardtii*’s ability to tolerate abiotic stresses. Considering that microalgae usually grow in water, and in order to determine the upper limit of the transformants’ tolerance to various stresses, we conducted a liquid TAP medium gradient stress experiment in a 24-well plate. The results showed that the growth of the transformants in the alkaline stress and oxidative stress group was better than WT, but the growth advantage of transformants in the saline-alkali stress and salt stress group was not significant compared to WT (Figure 2B). Next, we tested the growth dynamics of transformants and WT cultured in conical shake flasks to plateau under alkaline stress conditions. Under alkaline stress at pH = 9, the growth of transformants and WT almost stopped in the first two days, and the biomass of WT decreased slightly. On the third day, the transformants began to resume growth. The transformants showed higher biomass than WT during the next few days until reaching the plateau. The growth of transformants and WT at pH = 9.5 was similar to pH = 9, but the time of growth stagnation was longer. On the first day, the biomass of both transformants and WT dropped significantly, then the transformants began to recover on the third day, two days earlier than WT (Figure 2C).

Taken together, these results indicate that heterologous expression of ChbZIP1 can enhance the tolerance of *C. reinhardtii* CC-849 to abiotic stress, especially alkaline environment stress.

### 2.3. ChbZIP1 Enhances Photosynthesis of C. Reinhardtii Under Alkaline Stress

To further explore the role of ChbZIP1 in the resistance of microalgae to alkaline stress, transcripts of transformants (bZIP1-C16) and WT were obtained under alkaline stress conditions (pH = 9) for 0, 6, and 12 h, and their transcription characteristics at different time points were assessed. All genes and transcripts obtained by transcriptome assembly were compared, and each sample duplicate was well clustered together in the principal component analysis (PCA) graph, and the samples were correlated (Figure S2A). 1264, 509, and 1300 differentially expressed genes (DEGs) were upregulated and 1086, 160, and 944 differentially expressed genes (DEGs) were downregulated at hours 0, 6, and 12, respectively (Figure S2B,C). KEGG functional annotation analysis showed that the impact of ChbZIP1 on transformants was mainly concentrated at the level of cell metabolism (Figure S2D). The main functions of DEGs are distributed in energy, vitamin, carbohydrate, amino acid, and lipid metabolism. Genes and cellular processes such as translation, folding, and material transport are also distributed. We selected 10 DEGs for RT-qPCR verification, the results showed that the differences in the expression of these DEGs in transcripts and WT were consistent with the transcriptome results, proving the reliability (Figure S3).

**Figure 3:**
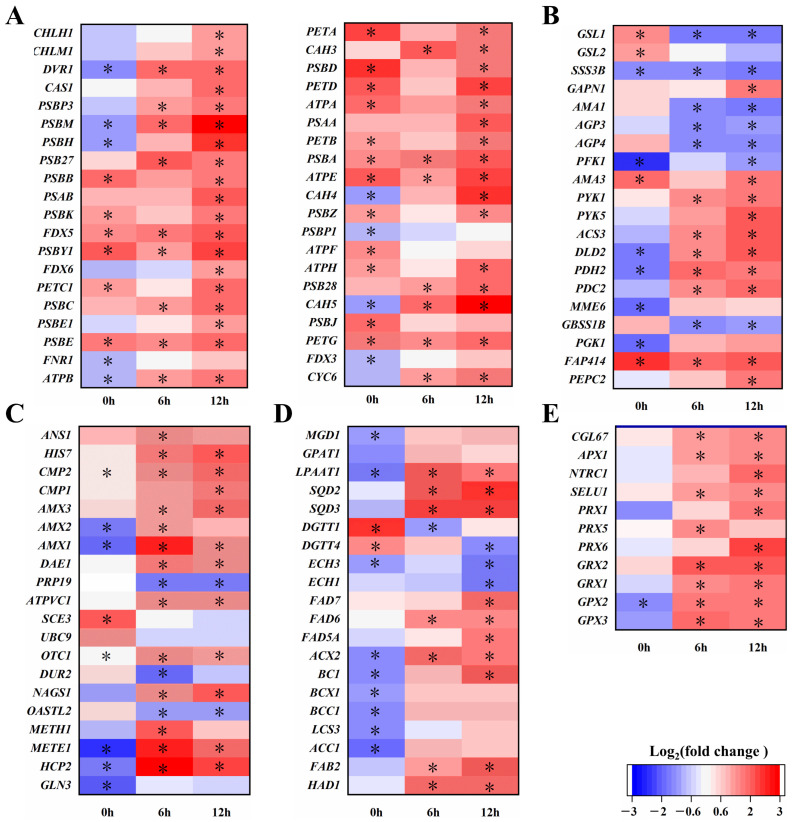
Differential gene expression of the selected metabolic pathways between the ChbZIP1-overexpressed transformant bZIP1-C16 and WT in an alkaline environment (pH = 9). Classified according to the different metabolic pathways involved in differentially expressed genes, divided into photosynthesis (**A**), carbohydrate metabolism (**B**), protein and amino acid metabolism (**C**), lipid metabolism (**D**), and ROS metabolism (**E**). Boxes with different color shades represent the multiple differential expression of genes. The right side of the figure is the legend. Significant differences are denoted by asterisks as absolute log_2_ (fold change) values > 1 and *p* < 0.05; *n* = 3.

Transcript data showed that genes related to photosynthesis were significantly upregulated under alkaline stress conditions (pH = 9) (Figure 3A). ChbZIP1 increases the expression of genes encoding photosystem I reaction center protein (*psaA*), photosystem II reaction center protein (*psbA*/*psbB*/*psbD*), cytochrome (*PETC1*/*CYC6*), oxygen evolution complex (*PSBP3*), electron transport chain (*petA*/*petD*/*FNR1*), and F-type ATP synthase (*atpA*/*atpB*), which may enhance the electron transport efficiency of bZIP1-C16, promoting photosynthesis. Genes related to porphyrin and chlorophyll metabolism (*CHLH1*/*CHLM1*/*DVR1*) have been upregulated. And we also found that genes related to carbonic anhydrase (*CAH5*/*CAH4*/*CAH3*), RuBisCO activating enzyme (*RCA1*), and bicarbonate transporter (*CAS1*), which are involved in the carbon dioxide concentration mechanism (CCM), were significantly upregulated.

**Figure 4:**
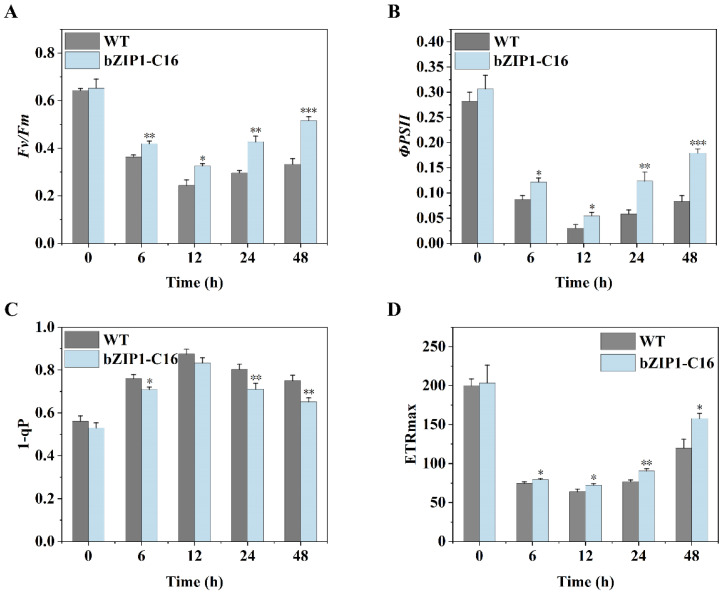
Comparison of the dynamic changes in the photochemical quenching parameters between the ChbZIP1-overexpressed transformant bZIP1-C16 and WT in an alkaline environment (pH = 9). *Fv*/*Fm* (**A**), ΦPSII (**B**), 1-qP (**C**), and ETRmax (**D**). They were first grown in a TAG medium at pH 7.0, then transferred to an AMPSO-acetate phosphate medium (pH = 9.0). The cells were collected at 0, 6, 12, 24, and 48 h of culture to determine the photochemical quenching parameters. Values are mean ± SD, (*n* = 3). Student’s *t*-tests were performed to determine statistically significant differences: *, *p* < 0.05; **, *p* < 0.01; and ***, *p* < 0.001.

To verify the effect of ChbZIP1 on the photosynthesis of transformants, we cultured bZIP1-C16 and WT under alkaline stress (pH = 9) and measured their photochemical quenching parameters at 0, 6, 12, 24, and 48 h, respectively (Figure 4). At the early stage of stress, both the maximum quantum yield of PSII (*Fv*/*Fm*) and the photochemical quantum yield of PSII (ΦPSII) decreased significantly. However, the values of bZIP1-C16 were significantly higher than WT (Figure 4A,B), which indicates that bZIP1-C16 had higher photosynthetic activity than WT under alkaline stress. Then, we measured the proportion of closed PSII reaction centers (1-qP) to reveal the degree of reduction in the plastoquinone for PSII electron transport. The results showed that WT had a higher proportion of closed reaction centers (Figure 4C), indicating that the electron transport chain in WT was more severely damaged. Further, we measured the maximum electron transport rate (ETRmax), which showed that bZIP1-C16 had a higher electron transfer rate than WT (Figure 4D), indicating that bZIP1-C16 had a higher photosynthetic affinity.

**Figure 5:**
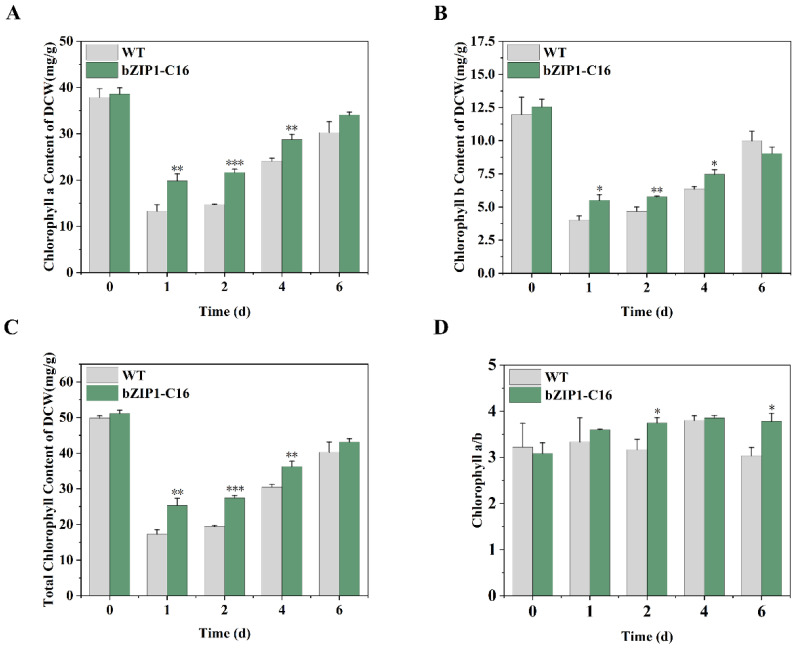
Comparison of the dynamic changes in the chlorophyll content of the ChbZIP1-overexpressed transformant bZIP1-C16 and WT in an alkaline environment (pH = 9). The light absorption value was measured to calculate the chlorophyll a content (**A**), chlorophyll b content (**B**), total chlorophyll content (**C**), and the ratio of chlorophyll a to b (**D**). They were first grown in a TAG medium at pH 7.0, and then transferred to an AMPSO-acetate phosphate medium (pH = 9.0). The cells were collected on 0, 1, 2, 4, and 6 days of culture, and chlorophyll was extracted with ethanol. Values are mean ± SD, (*n* = 3). Student’s *t*-tests were performed to determine statistically significant differences: *, *p* < 0.05; **, *p* < 0.01; and ***, *p* < 0.001.

The measurement of chlorophyll content further confirmed the above conclusion. Under alkaline stress (pH = 9), the chlorophyll a, chlorophyll b, and total chlorophyll contents of WT and bZIP1-C16 decreased, but the content of bZIP1-C16 was higher than that of WT (Figure 5A–C). This indicates that ChbZIP1 reduces the degradation of chlorophyll in *C. reinhardtii* under alkali stress. The chlorophyll a/chlorophyll b ratio of bZIP1-C16 was also higher than WT (Figure 5D), and combined with the changes in the total chlorophyll content, it showed that bZIP1-C16 had better photosynthesis efficiency, which was in line with the analysis of transcriptomics and photochemical quenching parameters.

The above results suggest that the expression of ChbZIP1 increases the photosynthesis efficiency of *C. reinhardtii* by upregulating the genes related to the electron transport chain, CCM, and photosynthetic pigment synthesis.

### 2.4. ChbZIP1 Affects the Cellular Composition of C. reinhardtii Under Alkaline Stress

**Figure 6:**
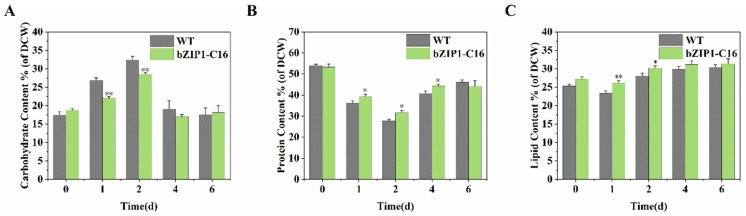
Comparison of the dynamic changes in the main biochemical components between the ChbZIP1-overexpressed transformant bZIP1-C16 and WT in an alkaline environment (pH = 9). The total carbohydrate content (**A**), total protein content (**B**), and total lipid content (**C**) were measured respectively. They were first grown in a TAG medium at pH 7.0, and then transferred to an AMPSO-acetate phosphate medium (pH = 9.0). The cells were collected on days 0, 1, 2, 4, and 6 of culture. Values are mean ± SD, (*n* = 3). Student’s *t*-tests were performed to determine statistically significant differences: *, *p* < 0.05; and **, *p* < 0.01.

Since enhanced photosynthesis leads to more CO_2_ being fixed, transcriptomic analysis also found that ChbZIP1 affects the expression of genes related to carbohydrate, protein, and lipid metabolism (Figure 3B–D). Thus, we measured the content of various cell components of bZIP1-C16 and WT under alkaline stress (pH = 9) on 0, 1, 2, 4, and 6 days to explore the effect of ChbZIP1 on the carbon flow distribution of *C. reinhardtii* under alkaline stress. The changes in cell components in bZIP1-C16 and WT were basically the same, with carbohydrate content increasing first and then decreasing, while protein and lipid content both decreased first and then increased. The difference is that bZIP1-C16 showed less carbohydrate accumulation, higher lipid accumulation, and less protein degradation during stress compared to WT (Figure 6).

The differences in cell composition were most significant on the 1st and 2nd days after the stress occurred, with the carbohydrate content of bZIP1-C16 being 18.1% and 11.9% lower than WT, respectively (Figure 6A). Combined with transcriptome data analysis, it was found that the bZIP1-C16 genes associated with starch synthesis (*GBSS1B*/*AGP3*/*AGP4)* were downregulated at 6 and 12 h. The genes related to TCA cycle and pyruvate metabolism (*PEPC2*/*DLD2*/*PDH2*/*ACS3*) were significantly upregulated (Figure 3B). The determination of protein content showed that on 1, 2, and 4 days, the protein content of bZIP1-C16 was 8.4%, 14.3%, and 8.7% higher than that of WT, respectively (Figure 6B). The expression of amino acid synthesis (*NAGS1*/*OTC1*/*CGLD*/*SHMT2*/*AMX*/*GLN3*) was significantly upregulated, while some degrading enzymes (*DUR2*/*OASTL2*) were significantly downregulated (Figure 3C). The lipid content of bZIP1-C16 was 12% and 7.7% higher than that of WT on 1 and 2 days under alkaline stress, respectively (Figure 6C). Analysis of transcriptome data found that in addition to some enzymes related to glycerol phospholipid and glycerol metabolism (*PGA2*/*PGA6*/*BTA1*) being upregulated, some fatty acid desaturases (*FAB2*/*FAD6*/*FAD7*/*HAD1*) and fatty acid chain elongation enzymes (*FAE3*) were also significantly upregulated (Figure 3D). Therefore, we further analyzed the fatty acid composition of bZIP1-C16 and WT at 0, 6, 12, 24, and 48 h under alkaline stress. The results showed that the C16:0, C16:1, and C18:0 contents of bZIP1-C16 decreased, while the C16:3, C18:1, C18:2, C18: 3n6, and C18: 3n3 contents increased significantly (Table 1). This shows that bZIP1-C16 has a decrease in saturated fatty acid (SFA) content compared with WT, with an increase in polyunsaturated fatty acid (PUFA) content, indicating that ChbZIP1 can increase the fatty acid unsaturation of *C. reinhardtii*.

The above results reveal that ChbZIP1 changes the carbon flow within *C. reinhardtii* cells, responding to the stressful environment by reducing the accumulation of carbohydrates and weakening protein degradation, thereby synthesizing more lipids and simultaneously increasing lipid unsaturation.

### 2.5. ChbZIP1 Enhances ROS Detoxification of C. reinhardtii Under Alkaline Stress

**Figure 7:**
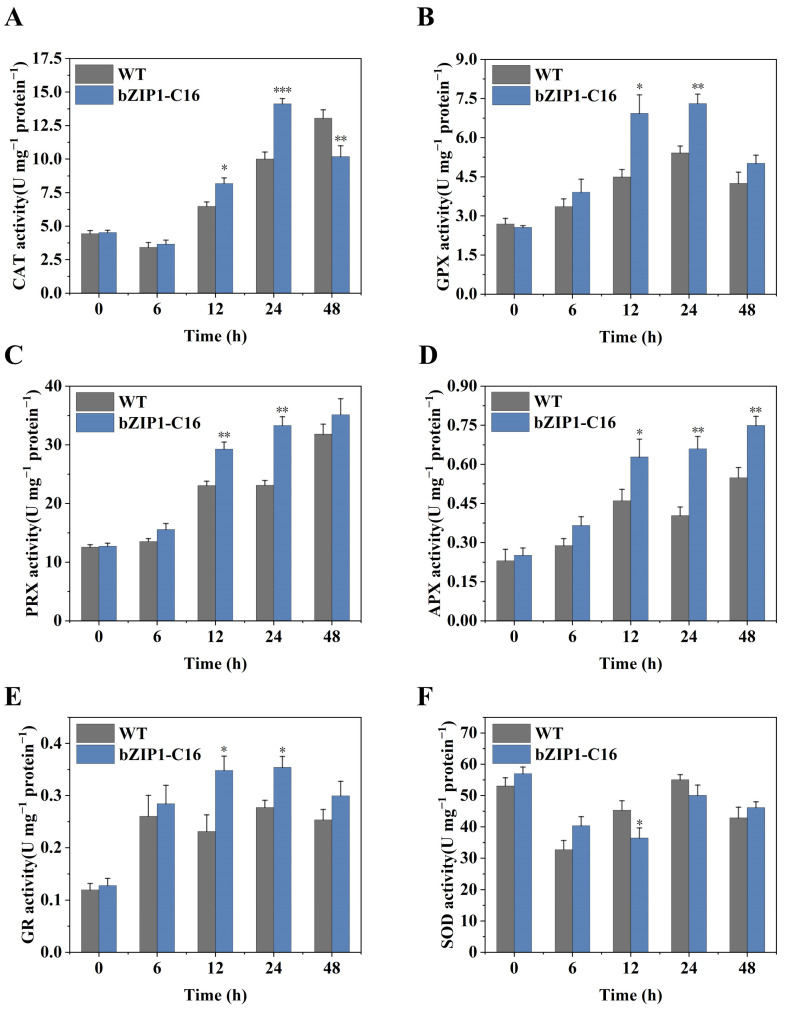
Comparison of the dynamic changes in antioxidant enzyme activities between the ChbZIP1-overexpressed transformant bZIP1-C16 and WT in an alkaline environment (pH = 9). The cells were collected at 0, 6, 12, 24, and 48 h of culture and the catalase (CAT) activity (**A**), glutathione peroxidase (GPX) activity (**B**), peroxidase (PRX) activity (**C**), Ascorbate peroxidase (APX) activity (**D**), glutathione reductase (GR) activity (**E**), and superoxide dismutase (SOD) activity (**F**) were measured using enzyme activity measurement kits. They were first grown in a TAG medium at pH 7.0, and then transferred to an AMPSO-acetate phosphate medium (pH = 9.0). Values are mean ± SD, (*n* = 3). Student’s *t*-tests were performed to determine statistically significant differences: *, *p* < 0.05; **, *p* < 0.01; and ***, *p* < 0.001.

Almost all stress environments cause the production of ROS inside plant cells, and more will be produced in alkaline stress [4]. If cells fail to detoxify these ROS in time, normal growth will be seriously affected, and more seriously, death will be caused. Through the analysis of transcriptome data, we found that bZIP1-C16 upregulated several genes (*GPX2*/*GPX3*/*GRX1*/*APX1*/*PRX5*/*PRX6*) related to the ROS detoxification process at 6 and 12 h under alkaline stress (Figure 3E). This suggests that ChbZIP1 may regulate these genes to reduce the ROS produced under stress to improve stress tolerance. To verify this conjecture, we tested the enzyme activities of enzymes involved in the ROS detoxification process of bZIP1-C16 and WT at 0, 6, 12, 24, and 48 h under alkaline stress conditions (pH = 9), as well as the relative abundance of ROS and the relative content of malondialdehyde (MDA) of bZIP1-A7, bZIP1-C16, and WT. The results showed that in bZIP1-C16, except for SOD, the enzyme activities of other antioxidant enzymes related to ROS clearance, like catalase (CAT), peroxidase (PRX), glutathione peroxidase (GPX), ascorbate peroxidase (APX), and glutathione reductase (GR), were significantly higher than those of WT after 12 h of stress (Figure 7).

**Figure 8:**
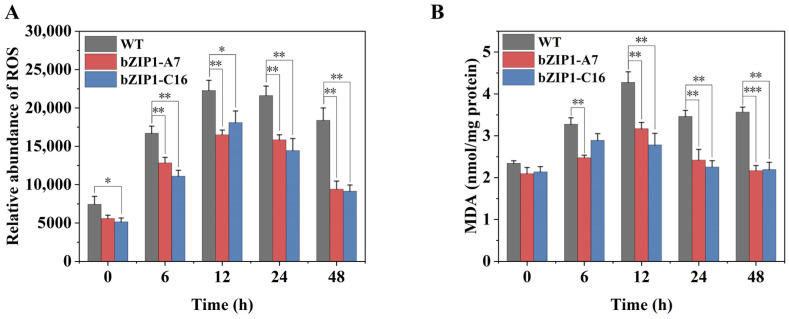
Comparison of the dynamic changes in the oxidative stress status indicators between the ChbZIP1-overexpressed transformants bZIP1-C16 and WT in an alkaline environment (pH = 9). The relative abundance of ROS (**A**) and MDA content (**B**) were measured using flow cytometry analysis and MDA detection kits, respectively. They were first grown in a TAG medium at pH 7.0, and then transferred to an AMPSO-acetate phosphate medium (pH = 9.0). The cells were collected at 0, 6, 12, 24, and 48 h of culture. Values are mean ± SD, (*n* = 3). Student’s *t*-tests were performed to determine statistically significant differences: *, *p* < 0.05; **, *p* < 0.01; and ***, *p* < 0.001.

The measurement of the relative intracellular ROS abundance revealed that ROS in WT and transformants increased rapidly at the beginning, with ROS in bZIP1-A7 and bZIP1-C16 peaking at 12 h and then decreasing, while ROS in WT remained at a high level (Figure 8A). The measurement of MDA content related to membrane lipid oxidation also showed that MDA accumulation in bZIP1-A7 and bZIP1-C16 was significantly lower than in WT under stress conditions (Figure 8B).

These results reveal that ChbZIP1 upregulate the expression of ROS detoxification enzyme genes to have a higher ROS detoxification ability to ensure survival under alkaline stress.

## 3. Discussion

There are a large number of saline-alkali lands, lakes, and wastewater that need to be developed and utilized around the world [1]. Some microalgae can grow in the above environments, but research on their alkali-resistant mechanisms is still lacking [37]. Here, we genetically modified *C. reinhardtii*, an excellent biological chassis with wide application prospects, through genetic engineering, which has successfully improved its adaptability in moderately alkaline environments.

### 3.1. ChbZIP1 Improves the Extensive Stress Resistance of C. reinhardtii, Especially the Alkali Resistance

The role of the bZIP transcription factor family in eukaryotes, especially higher plants, has been extensively studied. It has extensive effects on plant abiotic stresses and their regulatory mechanisms [38], but there are few reports on bZIP TFs in microalgae [39]. Therefore, we selected the ChbZIP1 TF of *Chlorella* sp. BLD, which can tolerate high pH values (pH > 10), as the research object and combined physiological and biochemical analysis, transcriptomics, molecular biological testing, and other methods to explore the possible mechanism of the ChbZIP1. We used sequence alignment and neighbor-joining (NJ) tree analysis to determine that ChbZIP1 belongs to the bZIP TFs family and has a certain homology with bZIP TFs from other microalgae and plants (Figure 1A,B). Then, by expressing the ChbZIP1-mVenus fusion protein in *C. reinhardtii*, we determined that it localized to the nucleus rather than the cytoplasm (Figure 1C), further demonstrating that it may perform the role of a transcription factor.

In previous reports, overexpressing soybean’s bZIP transcription factor GsbZIP67 was shown to confer better growth on alfalfa under 50 mM NaHCO_3_ treatment, as evidenced by longer roots and buds [40]. The overexpression of another bZIP TF SlAREB1 from tomato increases the antioxidant capacity of transgenic tomatoes under salt-alkali stress [41]. A recent study showed that the functional identification of the MhbZIP23 gene of Begonia shows that it can improve the alkali tolerance of Arabidopsis [42]. In this study, we also observed that the transgenic mutant strain bZIP1-C16 expressing the transcription factor ChbZIP1 had better growth than WT in an alkaline environment, manifested by a faster recovery cycle and higher biomass during the plateau period (Figure 2C). This may reveal that ChbZIP1 may, like other members of the bZIP family, play an important regulatory element role in the tolerance of algae or plants to saline-alkali environments. In addition, in the agar plate experiments, we also found that bZIP1-C16 not only performed better than WT in saline-alkali environments such as alkali, salt, and alkaline salts, but also grew better in environments such as high light and oxidative stress, even if not as pronounced as in saline-alkali environments (Figure 2A). Other bZIP TFs have also shown similar ability to respond extensively to various environmental stresses. The *IbbZIPs* gene from sweet potato is highly induced to be upregulated under various stresses [43], and the bZIP TF Yap1 is involved in resistance to multiple stresses, such as oxidative stress and virulence [44]. These results suggest that the ChbZIP1 may have a broader ability to regulate abiotic environmental stresses, but it is more significant in alkaline stress.

### 3.2. ChbZIP1 Enhanced Photosynthesis, Enhances Carbon Dioxide Concentration, Promotes Organic Matter Synthesis and Reduces ROS Production

Photosynthesis is the main way for plants and algae to obtain energy and is the source of power for their growth and survival [45]. Different environmental factors, such as high temperatures and heavy metals, have a negative impact on the photosynthetic system of cells, causing growth restrictions and, in serious cases, causing their death [46]. Similarly, an alkaline environment can also hinder photosynthesis, seriously threatening survival. Alkaline stress caused by high pH greatly downregulates the maximum quantum efficiency of photosystem II (PSII) and the quantum yield of electron transport in plant cells. At the same time, the chloroplast thylakoid membranes are also damaged [10]. We proved this by measuring photosynthetic parameters (Figure 4) and chlorophyll content (Figure 5) in an alkaline environment. Enhanced photosynthesis has been shown to effectively reduce damage to plant cells in adverse environments and improve the synthesis of cellular components such as carbohydrates and lipids [47]. Transcriptomic analyses reveal a significant upregulation of the genes associated with the components of the photosynthetic electron transport chain in bZIP1-C16 under alkaline conditions (Figure 3A). This finding suggests that ChbZIP1 may facilitate the restoration of normal photosynthetic activity in cells by rescuing compromised reaction centers involved in the photosynthetic process. We did detect enhanced photosynthetic activity (Figure 4) and more chlorophyll accumulation (Figure 5) in bZIP1-C16, suggesting that bZIP1-C16 has stronger photosynthesis than WT, thereby reducing cell damage caused by alkaline stress and providing more energy to synthesize cell components to support their growth. Notably, we found that CCM-related genes (*CAH3, CAH4, CAH5*, and *CAS1*) in bZIP1-C16 were upregulated in alkaline environments (Figure 3A). This indicates that ChbZIP1 may provide sufficient CO_2_ for the dark reaction of photosynthesis by enhancing CCM, promote the synthesis of organic matter, consume ATP and NADPH produced by the light reaction to create an electron collection pathway to further enhance photosynthesis, and thereby reduce the production of reactive oxygen species (ROS) [48].

### 3.3. ChbZIP1 Alters Carbon Flow Distribution to Synthesize Lipids and Increases Fatty Acid Unsaturation to Repair Cell Membrane Structure

In adverse environments, cells often undergo dramatic changes in their intracellular components in response to stress. Generally, plants and eukaryotic microalgae cells choose to weaken the synthesis of components related to growth and instead synthesize energy storage substances such as starch and glycerol, as well as substances such as transporters and antioxidant enzymes related to responding to stress [49,50,51]. Under stress conditions, lipids are the main energy storage substances for cells to survive. Lipid accumulation in *C. reinhardtii* has been shown to be related to salt concentration, and as salt concentration increases, lipid content increases [52]. Furthermore, the regulation of membrane lipid composition in response to various environmental conditions plays a critical role in plant stress adaptation. Notably, alterations in fatty acid composition proportions represent a primary response to the cell’s need for maintaining membrane fluidity [53,54]. Some reports on bZIP TF in microalgae reveal that they are related to the growth of algae cells and their lipid remodeling [55]. Another report has shown that overexpressing the bZIP TF can increase biomass accumulation and lipid productivity of *Nannochloropsis* under nitrogen deficiency and salt stress [56]. Consistent with these reports, both bZIP1-C16 and WT are more likely to accumulate energy storage substances such as carbohydrates and lipids in an alkaline environment. However, bZIP1-C16 tends to accumulate more lipids rather than carbohydrates compared to WT (Figure 6A,C). This observation is corroborated by transcriptomic data, which indicate the downregulation of the genes associated with starch biosynthesis (Figure 3B). Phosphatidylglycerol (PG) is a crucial component of Photosystem II (PSII). In high-salinity environments, augmenting the levels of unsaturated fatty acids (UFA) in membrane lipids has been shown to mitigate light-induced inhibition of PSII [57,58]. Additionally, it has been documented that increasing the unsaturation of PG can facilitate the repair processes of PSII [59]. In bZIP1-C16, the content of saturated fatty acids and some monounsaturated fatty acids decreases, while the content of polyunsaturated fatty acids increases (Table 1). Consequently, there is an upregulation of the genes associated with specific fatty acid desaturases and elongases (Figure 3D). The above results indicate that ChbZIP1 significantly affects the distribution of carbon flow within cells under alkaline conditions, making it more inclined to reduce the accumulation of carbohydrates and instead synthesize more lipids, increasing the unsaturation degree of fatty acids. This promotes the repair of cellular membrane structures, including the thylakoid membranes of chloroplasts.

### 3.4. ChbZIP1 Enhances the Detoxification Ability of ROS, Maintains Cell Homeostasis and Reduces Cell Structural Damage

Reactive oxygen species (ROS) are a part of normal cellular metabolism. However, under adverse environmental conditions, excessive production of ROS can lead to lipid peroxidation, protein oxidation, and the activation of programmed cell death (PCD) pathways, ultimately resulting in cell death [60]. In high-salinity environments, reactive oxygen species (ROS) accumulate in significant amounts, resulting in damage to the light-harvesting complex (LHC) of photosystem I (PSI) and the proteins of photosystem II (PSII). Concurrently, ROS also contributes to the disruption of various membrane structures within organelles as well as the cellular membrane [61]. bZIP TF slaREB1 plays a crucial role in the regulation of antioxidant defense mechanisms in tomato plants under salt-alkali stress. Its overexpression significantly enhances the activity of intracellular antioxidant enzymes, improving the cell’s capacity to scavenge excess ROS [41]. In this study, we identified several oxidative detoxification-related genes that may be upregulated by ChbZIP1 under alkaline conditions (Figure 3E). The results confirmed our inference, showing that the activity of ROS detoxifying enzymes (SOD, CAT, PRX, GPX, APX, and GR) in bZIP1-C16 cells is significantly higher than that in WT (Figure 7), while the relative abundance of ROS and the accumulation of MDA are significantly lower (Figure 8). As for the enzyme activity of SOD being lower than that of WT at 12 and 24 h (Figure 7A), we speculate that it may be because other ROS detoxification enzymes have already played a major role, allowing cells to complete ROS detoxification without a higher enzyme activity of SOD. These results indicate that ChbZIP1 can enhance the expression of the genes related to oxidative detoxification, thereby increasing the activity of antioxidant enzymes, eliminating excessive ROS, and mitigating potential damage to cellular structures.

## 4. Materials and Methods

### 4.1. Microalgae Strains and Cultivation

The *C. reinhardtii* strain used in this study was purchased from the Chlamydomonas Resource Center at the University of Minnesota and numbered CC-849 (cw10mt-). The algae strain was cultured using a Tris-Acetate-Phosphate (TAP) medium. The temperature was 25 ± 1.5 °C, the light intensity was 100 μmol photons m^−2^s^−1^, and the volumes of the conical flasks were 250 mL and 500 mL. Cells were cultured under continuous white light irradiation and shaken at 120 rpm.

### 4.2. Construction and Verification of C. reinhardtii Transformants

In order to increase the expression of transcription factors in *C. reinhardtii*, the coding sequence of ChbZIP1 was codon-optimized, and an intron was inserted between two guanines separated by about 500 bp [35], and then used to replace the mVenus fragment on plasmid pMO449. For the subcellular localization vector, ChbZIP1 without the stop codon was inserted before the mVenus fragment on pMO449. The plasmid was linearized using the restriction endonuclease EcoRV, purified, and recovered, and transformed into *C. reinhardtii* CC-849 cells using a glass bead agitation method. After transformation, the cells were plated on paromomycin-resistant plates for screening for positive transformants. The genomic DNA of the positive transformants was extracted using Hexadecyltrimethylammonium bromide (CTAB). The total RNA was extracted using the R6870 Yeast RNA Kit (Yeasen, Shanghai, China). The first strand of cDNA was synthesized using the Hifair^®^ III 1st Strand cDNA Synthesis SuperMix Kit (Yeasen, Shanghai, China). Polymerase chain reaction (PCR) was performed using amplification primers (Table S1) to verify the integration of the ChbZIP1 gene in the genome and whether it could be expressed in cells and correctly cut the intron.

### 4.3. Subcellular Localization Assay

To observe the subcellular localization of the ChbZIP1 transcription factor, the transformed transformants expressing the ChbZIP1_mVenus fusion protein were cultured. Take 5 µL of the algae solution on a glass slide, cover it with a coverslip, place it in the sample tank of a confocal laser scanning microscope (Nikon, Minato, Japan), use an 80× eyepiece, and select the FITC, Cy5, and TD channels to observe fluorescence. mVenus fluorescence is excited at 488 nm and signals are collected at 539–591 nm. Chlorophyll autofluorescence is excited at 640 nm and signals are collected at 620–700 nm.

### 4.4. Transformants Growth Assay

To create alkaline stress conditions, Tris in TAP medium was replaced with 25.6 mM of 3-[(1,1-Dimethyl-2-hydroxyethyl) amino]-2-hydroxypropanesulfonic acid (AMPSO) and N-Cyclohexyltaurine (CHES), respectively, and the pH of the medium was adjusted to 9, 9.5, 9.65, 9.7, and 9.8 with potassium hydroxide (KOH). For alkaline salt stress conditions, 16, 24, and 32 mM NaHCO_3_ were added to the TAP medium, respectively. For salt stress conditions, 150, 250, and 350 mM NaCl were added to the TAP medium, respectively. For oxidative stress conditions, 2.5 mM metronidazole (Met) or 50 μmol tert-Butyl hydroperoxide(tBOOH) was added to the TAP medium. While preparing the agar plate, 1% agar was added based on the above methods. On solid plates, the algae strains were cultured until the OD_750_ value was 1.2. After gradient dilution, 10 µL drops were taken and placed in the grid of a square Petri dish (10 × 10 cm), inverted, and cultured under light conditions. In 24-well plates, the algae strain was cultured until the OD_750_ value was 1.2, and then inoculated into a 24-well plate liquid culture medium with different levels of stress, and cultured under light conditions. In flasks, the algae strain was cultured to an OD_750_ value of 1.2, and then inoculated into 250 mL conical flasks containing 100 mL TAP medium at pH = 9 and 9.5, and cultured under light conditions at 120 rpm.

### 4.5. RNA Extraction, Transcriptome Sequencing and Analysis

According to the above culture conditions, ChbZIP1 transformants and WT algae strains were cultured, respectively, under the alkaline stress conditions of pH = 9. Microalgae cells were collected by centrifugation (Eppendorf, Hamburg, Germany) at 0, 6, and 12 h and quickly frozen using liquid nitrogen. The purification and isolation of total RNA from microalgae, quality evaluation of RNA, and sequencing of high-quality RNA were completed by Majorbio (Shanghai Majorbio BioPharm Technology, Shanghai, China). The resulting transcriptome data were also analyzed by Majorbio. The corresponding raw data from RNA sequencing were deposited in the Genbank repository with the accession number PRJNA974905. Its |log_2_FC| Genes with ≥1 and *p*-adjustment < 0.05 are considered differentially expressed genes (DEGs).

### 4.6. Verification of Relative Gene Expression Levels by RT-qPCR

cDNA was synthesized using the Hifair III First Strand cDNA Synthesis SuperMix (Yeasen, Shanghai, China) according to the manufacturer’s instructions. All the primers used for qPCR are outlined in Table S1. qPCR analysis was performed on a qTOWER3G Touch (Analytik Jena, Jena, Germany) using SuperReal PreMix Plus (Tiangen, Beijing, China). The activation of the protein kinase C receptor (CBLP) (Cre06.g278222) was used as an internal control, and the relative expression levels were determined using the 2^−ΔΔCT^ method.

### 4.7. Quantitative Analysis of Main Components

According to the above culture conditions, ChbZIP1 transformant bZIP1-C16 and WT were cultured, respectively, under the alkaline conditions of pH = 9. The cells were collected by centrifugation (Eppendorf, Hamburg, Germany) on days 0, 1, 2, 4, and 6, and the cells were lyophilized using a vacuum freeze dryer (Labconco Kansas, Kansas, MI, USA), and then stored in a dry storage box for testing. Methods for determining total carbohydrate, total lipid, and total protein content were based on our previous reports [32]. The method to determine the chlorophyll content was to add 4.5 mL of ethanol and 240 mg of glass sand to 0.01 g of freeze-dried algae powder. The samples were homogenized with a grinding machine (Jingxin, Shanghai, China) in darkness for 3 min, repeated 3 times. The samples were centrifuged at 18,514× *g* for 5 min. Supernatants were diluted and the absorbance was measured at 632, 649, 665, and 696 nm. The method for calculating chlorophyll a and b content referred to the published method [62].

### 4.8. Determination of Photosynthetic Activity Index

According to the above culture conditions, ChbZIP1 transformants and EV strains were cultured, respectively, under the alkaline stress conditions of pH = 9. The algae liquor was collected at 0, 6, 12, 24, and 48 h and diluted to OD_750_ = 0.1 using the TAP medium. Various photosynthetic activity parameters (*Fv*/*Fm*, ΦPSII, qP, and ETRmax) were directly measured and recorded using a phytoplankton classification fluorometer (Heinz Walz, Effeltrich, Germany).

### 4.9. Determination of Antioxidant Enzyme Activities and Antioxidant Physiological and Biochemical Indicators

According to the above culture conditions, ChbZIP1 transformants and empty algae strains were cultured, respectively, under the alkaline stress conditions of pH = 9. Microalgae cells were collected by centrifugation (Eppendorf, Germany) at 0, 6, 12, 24, and 48 h; the supernatant was removed; and the algae cells were resuspended in PBS buffer, which was repeated three times. The resuspension was disrupted using a cell disruptor (SHUNMATECH, Nanjing, China) and then centrifuged to collect the supernatant. The protein concentration in the supernatant was determined using the BCA method. The enzyme activities of CAT, APX, GPX, PRX, GR and SOD were measured using Catalase (CAT) Activity Assay Kit, Ascorbate Peroxidase (APX) Activity Assay Kit, Cell Glutathione Peroxidase (GPX) Activity Assay Kit, Peroxidase (POD) Activity Assay Kit (Plant Samples), Glutathione Reductase (GR) Activity Assay Kit and Total Superoxide Dismutase (T-SOD) Activity Assay Kit (Hydroxylamine Method), respectively (Elabscience, Houston, TX, USA).

### 4.10. Statistical Analysis

All the experiments were conducted with three biological replicates, and the results are expressed as mean ± standard deviation (SD). Statistical analysis was performed using GraphPad Prism 9 and Origin 2018, and *p*-values were calculated using Student’s *t*-test.

## 5. Conclusions

**Figure 9:**
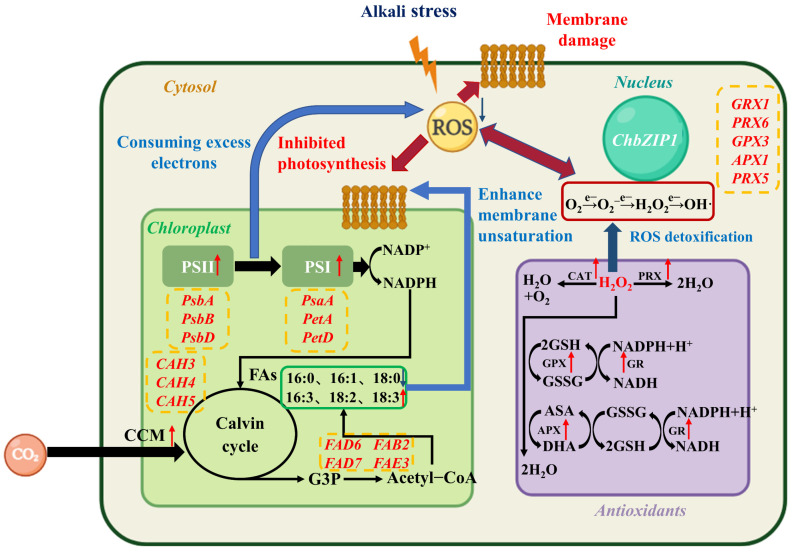
A proposed working model for ChbZIP1 regulating the tolerance of *Chlamydomonas reinhardtii* under an alkaline environment. ChbZIP1 repairs damaged electron transport chains and increases the supply of carbon dioxide by enhancing CCM, which enhances photosynthesis to synthesize more organic matter and provide energy while reducing excitation pressure on the photosynthetic electron transport chains, thereby inhibiting the production of ROS. ChbZIP1 enhances lipid synthesis and fatty acid unsaturation, promoting the repair of membrane structure and further repairing damaged photosynthesis. Additionally, ChbZIP1 enhances the expression levels of antioxidant enzymes, bolstering the clearance of excessive ROS. Red arrows represent increased product or enhanced pathway. Blue arrows represent decreased product or decelerated pathway.

This study enhanced the tolerance of *C. reinhardtii* CC-849 to alkaline environments through genetic engineering, resulting in superior growth and higher biomass accumulation. Next, the role of ChbZIP1 in regulating the alkali resistance of microalgae was verified, further revealing the possible alkali resistance regulatory network (Figure 9).

ChbZIP1 enhances the alkali tolerance of transformants by upregulating genes related to photosynthesis, cell component metabolism, and antioxidant effects. The enhancement of photosynthesis enables cells to synthesize more organic substances and provides more energy to adapt to alkaline environments. Concurrently, enhanced photosynthesis consumes excess electrons, creating an electron sink pathway that reduces ROS production. The accumulation of carbohydrates is weakened, leading to a diversion of excess carbon flow toward lipid synthesis, resulting in the production of a higher proportion of unsaturated fatty acids. Increased membrane lipid unsaturation enhances the integrity and repair of cellular and organelle membranes, thereby promoting the efficiency of photosynthesis. Moreover, the upregulation of antioxidant enzyme-related gene expression leads to an increase in the enzymatic activity of various antioxidant enzymes, enhancing the cells’ ability to detoxify ROS.

The above work provides a new direction and available elements for the genetic engineering transformation of plants related to alkali tolerance, and also provides new ideas and theoretical basis for the industrial utilization of alkali-resistant algae strains and the treatment of saline-alkali wastewater.

## Figures and Tables

**Table 1:** Comparison of the fatty acid composition between the ChbZIP1-overexpressed transformant bZIP1-C16 and WT in an alkaline environment (pH = 9). Values are mean ± SD, (*n* = 3). Student’s *t*-tests were performed to determine statistically significant differences: *, *p* < 0.05; **, *p* < 0.01; and ***, *p* < 0.001.

Fatty Acid (%)	0 h		6 h		12 h		24 h		48 h	
	WT	bZIP1-C16	WT	bZIP1-C16	WT	bZIP1-C16	WT	bZIP1-C16	WT	bZIP1-C16
C14:0	0.37 ± 0.03	0.26 ± 0.02 **	0.3 ± 0.06	0.27 ± 0.02	0.41 ± 0.03	0.40 ± 0.04	0.58 ± 0.02	0.45 ± 0.04 *	0.52 ± 0.02	0.47 ± 0.02
C16:0	34.61 ± 1.28	38.94 ± 1.81	40.49 ± 1	35.17 ± 0.82 **	39.8 ± 0.69	33.54 ± 0.49 ***	33.5 ± 0.1	28.31 ± 0.62 ***	34.3 ± 0.69	29.85 ± 0.6 **
C16:1	2.66 ± 0.25	3.17 ± 0.07 *	4.3 ± 0.23	3.86 ± 0.06	4.05 ± 0.18	3.08 ± 0.17 **	3.68 ± 0.19	2.95 ± 0.14 *	3.88 ± 0.08	2.53 ± 0.05 ***
C16:2	1.12 ± 0.03	0.79 ± 0.03 ***	1.45 ± 0.08	1.62 ± 0.11	0.74 ± 0.04	0.7 ± 0.06	1.03 ± 0.05	0.93 ± 0.03	1.03 ± 0.05	1.35 ± 0.11 *
C16:3n6	0.60 ± 0.06	0.45 ± 0.01 **	1.03 ± 0.04	1.2 ± 0.15	0.56 ± 0.02	0.54 ± 0.02	0.6 ± 0.02	0.46 ± 0.04 **	0.55 ± 0.01	0.61 ± 0.05
C16:3n3	1.06 ± 0.03	1.09 ± 0.02	1.77 ± 0.22	2.54 ± 0.08 **	2.45 ± 0.14	3.32 ± 0.17 **	3.54 ± 0.38	4.52 ± 0.07 *	2.46 ± 0.11	2.25 ± 0.08
C16:4	11.04 ± 0.51	10.21 ± 0.25	6.39 ± 0.52	6.19 ± 0.37	4.82 ± 0.26	5.8 ± 0.85	9.5 ± 0.42	9.56 ± 0.53	9.72 ± 0.31	10.03 ± 0.63
C18:0	8.21 ± 0.35	8.42 ± 0.34	9.93 ± 1.16	7.72 ± 0.48	10.77 ± 0.21	8.82 ± 0.17 ***	9.55 ± 0.31	8.04 ± 0.19 **	8.75 ± 0.19	6.79 ± 0.14 ***
C18:1	5.59 ± 0.09	4.49 ± 0.17 **	6.37 ± 1.05	7.02 ± 0.53	6.24 ± 0.56	7.19 ± 0.43	5.31 ± 0.06	7.5 ± 0.26 ***	5.85 ± 0.16	7.26 ± 0.41 *
C18:2	7.00 ± 0.32	5.74 ± 0.43 *	3.79 ± 0.15	5.44 ± 0.09 ***	3.47 ± 0.3	4.67 ± 0.45 *	3.75 ± 0.17	5 ± 0.45 **	4.67 ± 0.14	6.88 ± 0.32 ***
C18:3n6	3.83 ± 0.21	3.20 ± 0.07 *	3.1 ± 0.17	4.7 ± 0.33 **	4.09 ± 0.13	4.36 ± 0.3	4.53 ± 0.38	5.17 ± 0.11	4.43 ± 0.3	5.97 ± 0.54 *
C18:3n3	22.35 ± 0.53	21.70 ± 0.46	19.36 ± 1.08	22.4 ± 0.31 *	19.75 ± 0.54	24.55 ± 0.59 **	21.81 ± 0.47	23.63 ± 0.35 *	20.04 ± 0.25	22.93 ± 0.65 **
C18:4	1.56 ± 0.1	1.55 ± 0.08	1.71 ± 0.41	1.88 ± 0.09	2.86 ± 0.06	3.02 ± 0.11	2.61 ± 0.03	3.47 ± 0.05 ***	3.81 ± 0.19	3.08 ± 0.07 **
SFA	43.18 ± 1.51	47.62 ± 0.75 *	50.72 ± 2.02	43.16 ± 0.6 **	50.97 ± 0.83	42.77 ± 0.58 ***	43.64 ± 0.42	36.8 ± 0.57	43.56 ± 0.84	37.11 ± 0.6
MUFA	8.25 ± 0.09	7.66 ± 0.07	10.67 ± 1.26	10.87 ± 0.57	10.29 ± 0.4	10.27 ± 0.58	8.99 ± 0.22	10.46 ± 0.34	9.73 ± 0.23	9.78 ± 0.4
PUFA	48.56 ± 1.41	44.72 ± 0.34 *	38.61 ± 2.07	45.97 ± 0.49 **	38.74 ± 0.74	46.96 ± 0.32 ***	47.37 ± 0.54	52.74 ± 0.52 *	46.71 ± 0.66	53.11 ± 1 *

## Data Availability

Data are contained within the article and Supplementary Materials.

## Supplementary Materials

The following supporting information can be downloaded at: https://www.mdpi.com/article/10.3390/ijms26020769/s1.
