# Supplementary material for: Mechanism of Transcription Factor ChbZIP1 Enhanced Alkaline Stress Tolerance in Chlamydomonas reinhardtii
Source: Int J Mol Sci. 2025 Jan 17;26(2):769. doi: 10.3390/ijms26020769 (PMC11766021; doi:10.3390/ijms26020769)
Supplement: Supplementary file 1 [file ijms-26-00769-s001.zip › ijms-3400560-supplementary.pdf]

Figure S1. PCR verification of RNA reverse transcription cDNA (left) and genomic DNA (right) of algal strains of pMO-ChbZIP1.

1~4, bZIP1-D3, 12, 28, 30; 5~6, bZIP1-A7, 12; 7~8, bZIP1-B10, 24; 9, bZIP1-C16; 10, WT; M, Marker

Figure S2. Analysis of differentially expressed genes in bZIP1-C16 under alkaline environment (pH=9). (A) Principal Component Analysis (PCA) of the dataset. (B) Venn diagram of upregulated differentially expressed genes (DEGs). (C) Venn diagram of downregulated differentially expressed genes (DEGs). (D) KEGG annotation analysis of all time-point differentially expressed genes.

Figure S3. qPCR verification of differentially expressed genes in the bZIP1-C16 transcriptome under alkaline environment (pH=9).

CAH3, carbonic anhydrase; PSAA, Photosystem I P700 chlorophyll a binding protein A; PSBA, Photosystem II protein A; CYC6, cytochrome c6; PETA, cytochrome b6f complex; AGP3, ADP-glucose pyrophosphorylase; DUR2, histidase; DGTT1, diacylglycerol acyltransferase 1; FAD6, FAD-dependent acyl-CoA reductase; PRX6, chloroplast thiol-dependent peroxidase.

Data S1. Data for all statistical analyses

Data S2. The mRNA-Seq data for the genes involved in metabolism.

Table S1. Primers used in this study

Primer	Sequence
G-bZIP1-F	TCTGACGTCATGAGCGACGACG
G-bZIP1-R	ACCGCCACCACCGTCCTGCTGGTAGTGGTCGGC
Full-length-F	GATAAACCGGCCAGGGGGCCT
Full-length-R	CTCTCGTCCCCACCACCCTCCA
Sequence validation-F	GCAGTACCAGAAGAGCGGCGAGAAC
Sequence validation-R	ACCCACTCACAACCGGGATACCGAC
bZIP1-qPCR-F	GCAAGCAGAGCAACCGCGAGA
bZIP1-qPCR-R	GCGCCCTGAACGGCAGCATG
CAH3-qPCR-F	ACTATTTGTCTAATTTAATAAC
CAH3-qPCR-R	TGATCGTAATCCTGTAGAAACA
PSAA-qPCR-F	GGAAGCGTATGAGGATCGCA
PSAA-qPCR-R	CTGGTAGGAAGGTGGCGATG
PSBA-qPCR-F	TAGCTGCTTGGCCGGTAATC
PSBA-qPCR-R	ACGCTCGTGCATTACTTCCA
CYC6-qPCR-F	CCAGGTCTTCAACGGCAACT
CYC6-qPCR-R	GGGGATGTCCCGACCTGATA
PETA-qPCR-F	AACCCACGTGAGGCTAATGG
PETA-qPCR-R	TAATTTCTGCCGGAACGCGA
AGP3-qPCR-F	GCGACTGAGGAGGATTGAGG
AGP3-qPCR-R	GCGGCTTCTCCACATAGTCC
DUR2-qPCR-F	CTCCCCTCGACCAGCTTTTG
DUR2-qPCR-R	GATACCGCCCGCTTCAAGTA
DGTT1-qPCR-F	CGCTCACAATTGCCTTCCTG
DGTT1-qPCR-R	CCAGTCGGAAATGGCGATGA
FAD6-qPCR-F	TCCGTTCACTCTGCAAGACC
FAD6-qPCR-R	GACCACAAACAGAGCCCAGA
PRX6- qPCR-F	GCAGCCCATTGTTCTGTTCTT
PRX6-qPCR-R	GTCTTGCGCAGGATGGAGTT
CBLP- qPCR-F	GACGACCTGCGCCCCGAGTT
CBLP- qPCR-R	AGGCGCGGCTGGGCATTTAC

Table S2. RT-qPCR analysis of differentially expressed genes between bZIP1-C16 mutant and wild-type *Chlamydomonas reinhardtii* under alkaline stress

Gene ID	Gene name and time point	log_2FC	-ΔΔCT
Cre09.g415700_4532.v6.1	CAH3-6h	1.51359	1.86623
CreCp.g802280_4532.v6.1	PSAA-12h	1.84969	1.89720
CreCp.g802321_4532.v6.1	PSBA-6h	1.83525	2.01007
Cre16.g651050_4532.v6.1	CYC6-12h	1.46051	1.62530
CreCp.g802263_4532.v6.1	PETA-0h	2.0602	1.92380
Cre07.g331300_4532.v6.1	AGP3-6h	-1.39819	-1.8081
Cre08.g360100_4532.v6.1	DUR2-6h	-1.65393	-1.9206
Cre12.g557750_4532.v6.1	DGTT1-0h	2.29507	2.13786
Cre13.g590500_4532.v6.1	FAD6-12h	1.35898	1.64129
Cre10.g422300_4532.v6.1	PRX6-12h	2.10942	2.32988

Figure S1.

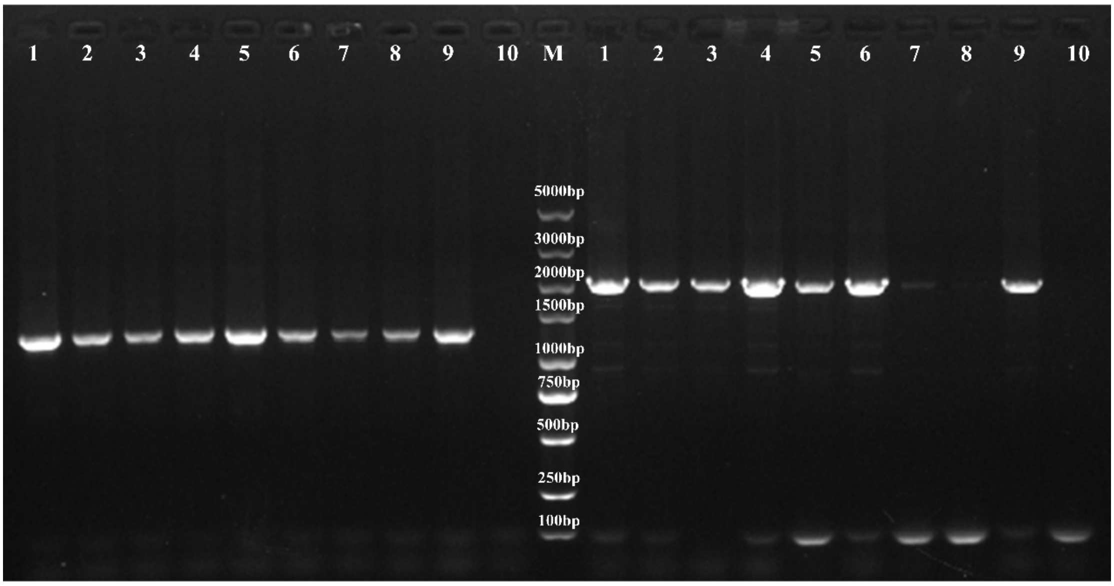

Figure S2.

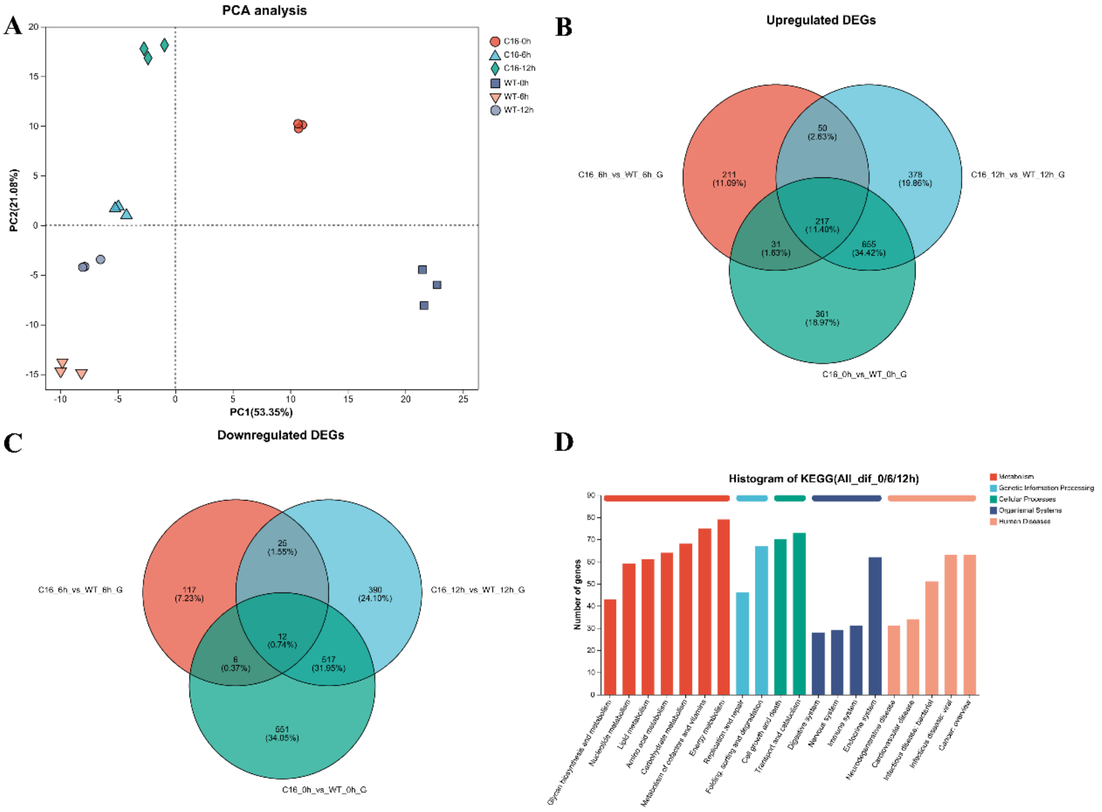

Figure S3.

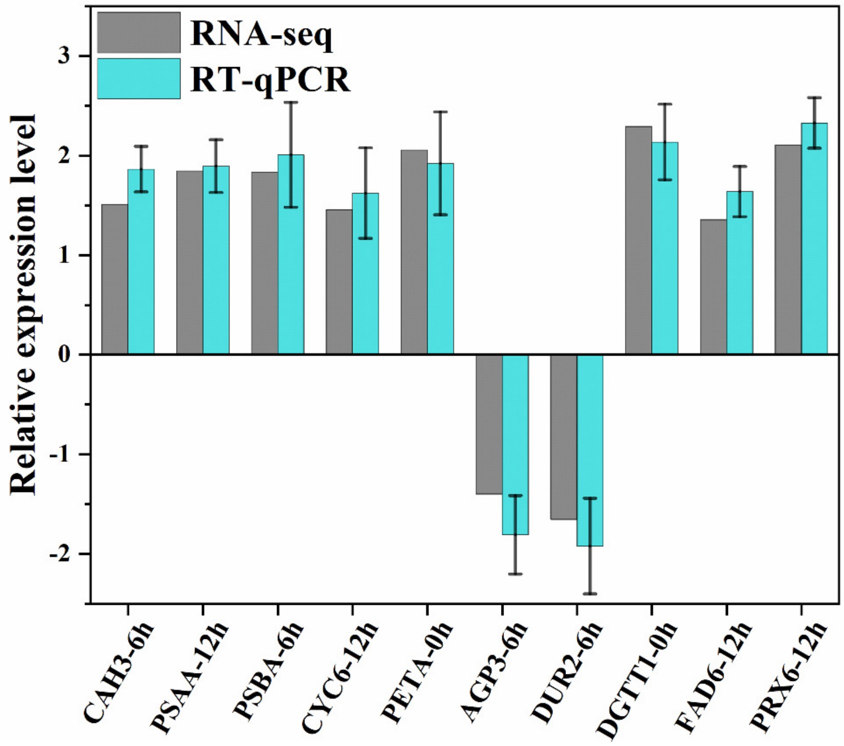
RNA-seq
RT-qPCR
Relative expression level
3
2
1
0
-1
-2
CAH3-6h
PSAA-12h
PSBA-6h
CYC6-12h
PETA-0h
AGP3-6h
DUR2-6h
DGTT1-0h
FAD6-12h
PRX6-12h

> Coding sequence of ChbZIP1(1191 bp)

ATGTCAGACGACGCTGCCGGAAAGGCTGACACCGTCGCGAAAGAAACGGAGGGC
AATGGGACGACCGCGGGAAATGCCCCCGGCTGTTCCTGGGAGGACATCATGAAAC
AATACCAGAAATCGGGAGAAAATGCAGGGACCAACGCTGGTTATATGCCCGGCAT
GTCTTTTTTCCCCACGGCCTTCATGGGCATGATGCCGTCCATGATGCCCTTCACTAG
CATGCCTTTCTTTCCCAACACCGGTGCTGGCGGAGGCTCAGAGGCAATTGCTGAA
GCAAAGGACGACGGTGCGAAACCTGCCGTTGAGGACGACGACGCCACGGAGGAT
GCTGATATAGGCGCCTCCGGGCCTTCTGCCGAAGTGGACACGGCTCAAGGTGGTA
AACGCACTGCAGCAGAAGCCGATGCAAATGGCAAACCCAACAAGCGGGCCAAAG
CAAGCAAAGTAGGCAAGGGTTTGGCAGTGAAGAGCAACAGCGATGCCTCGTTGG
CGCTGTTGGCATCTACAGCAAACCAAGCAACGATGCAAAATCCTATGCTAAACCTC
ATGAACGAGGTCCAACGCATGAACGAGATGCTCGGATATGCAAGCCCCACTGTTC
TTGGTGCTAATGCTGGTGGAGGTGGGGGCCTTGCAGGGACAGCAAACCCGCTCTC
TAATTCTGCTCCCAAGTCAGATAGCGAGCCACACGCAGGGACGGCAGGTCGTCGA
GCATCGGCAGGCACATCGGGTATTGATGATGCCGCTGAATTTGAGATAGATGAGGC
ATTGCTTGCGACCATGGACGAAAAAGACTTGAAGAAGCTTCGACGCAAACAATCA
AATCGTGAGAGTGCGCGGCGATCGCGATTGCGCAAACAGGCTGAAATAGAGCAGT
TGCAAGCGGAAAACCAAGCACTCCAGGGCGAGGTGCAGCAACTTCGGAATGAAA
AAGTGGAATTAAACGCACAAATTGCCATTTTGCACGCCAAGCTTAGCATGTCCTCT
GCATTTGGAGCATTAAATGGGTCCATGGCAGCTCTATCCCACCCAGGGGCCTCTAC
TGCCGCTGCGTTCCCGGTGTCTACTTCTTTGCCGGCTCCTGGGTCAGGTGCTGCCG
CTGTCGGTGGTGGTGGAGCAGACGCCAAGACGGACCAAGATAAGGTAAACACTG
CTACCGCGAGTGCAGCAGACCATTACCAACAATAA

> Optimized codons of ChbZIP1, containing intron sequences from *Chlamydomonas reinhardtii*, with the yellow high-lighted sections-1665 bp

ATGAGCGACGACGCCGCCGGCAAGGCCGACACCGTGGCCAAGGAGACGGAGGGC
AACGGCACCACCGCCGGCAACGCCCCCGGCTGCAGCTGGGAGGACATCATGAAG
CAGTACCAGAAGAGCGGCGAGAACGCCGGCACCAACGCCGGCTACATGCCCGGC
ATGAGCTTCTTCCCGACCGCCTTCATGGGCATGATGCCGAGCATGATGCCGTTCAC
CAGCATGCCCTTCTTCCCCAACACCGGCGCCGGCGGCGGCAGCGAGGCCATCGCC
GAGGCCAAGGACGACGGCGCCAAGCCCGCCGTGGAGGACGACGACGCCACCGA
GGACGCCGACATCGGCGCCAGCGGCCCCAGCGCCGAGGTGGACACCGCCCAGGG
CGGCAAGCGCACCGCCGCCGAGGCCGACGCCAACGGCAAGCCCAACAAGCGCGC
CAAGGCCAGCAAGGTGGGCAAGGGCCTGGCCGTGAAGAGCAACAGCGACGCCA
GCCTGGCCCTGTTGGTGAGTCGACGAGCAAGCCCGGCGGATCAGGCAGCGTGCTT
GCAGATTTGACTTGCAACGCCCGCATTGTGTCGACGAAGGCTTTTGGCTCCTCTGT
CGCTGTCTCAAGCAGCATCTAACCCTGCGTCGCCGTTTCCATTTGCAGGCCAGCAC
CGCCAACCAGGCCACCATGCAGAACCCCATGCTGAACCTGATGAACGAGGTGCAG
CGCATGAACGAGATGCTGGGCTACGCCAGCCCCACCGTGCTGGGCGCCAACGCCG
GCGGCGGCGGCGGCCTGGCCGGCACCGCCAACCCCCTGAGCAACAGCGCCCCCA
AGAGCGACAGCGAGCCCCACGCCGGCACCGCGGGCCGCCGCGCCAGCGCCGGCA
CCAGCGGCATCGACGACGCCGCGGAGTTCGAGATCGACGAGGCCCTGCTGGCCAC
CATGGACGAGAAGGACCTGAAGAAGCTGCGCCGCAAGCAGAGCAACCGCGAGA
GCGCCCGCCGCAGCCGCCTGCGCAAGCAGGCCGAGATCGAGCAGCTGCAGGCCG
AGAACCAGGCCCTGCAGGGCGAGGTGCAGCAGCTGCGCAACGAGAAGGTGGAG
CTGAACGCGCAGATCGCCATCCTGCACGCCAAGCTGAGCATGAGCAGCGCCTTCG
GCGCCCTGAACGGCAGCATGGTGAGCTTGCGGGGTTGCGAGCAACACTCCAGCA
ACGAACAGTGCCCAAGTCAGGAATCTGCAGTCAGCCTGGGCTTTCGGCGGCTTTT
TCTTGGGCAAACAGCTTGCACTCATGCCAGCGCGGCTTGTCCAGCCTCACTTGAG
CTTTCCAGCTGCTACCAGCCGGGCTATACGACAGCGACAGAGCCATAGCGTGGAAT
CACTTATTTGGGTTGCCGAAGTAGCGGTCGGAGCGTGAGTTCTTGGTCAAGCCGC
CCCTTATCCGGTTCCTGTCCGTGTCTTTGTCCCTCGTTCACCCTTCGCGGCACCCTT
CATCCCCTTGCTTGCAGGCCGCCCTGAGCCACCCCGGCGCCAGCACCGCGGCCGC
CTTCCCCGTGAGCACCAGCCTGCCCGCCCCCGGCAGCGGCGCCGCCGCCGTGGGC
GGCGGCGGCGCCGACGCCAAGACCGACCAGGACAAGGTGAACACCGCGACCGCC
AGCGCCGCCGACCACTACCAGCAGGAC
